# Protective Immunity Elicited by Oral Immunization of Mice with *Salmonella enterica* Serovar Typhimurium Braun Lipoprotein (Lpp) and Acetyltransferase (MsbB) Mutants

**DOI:** 10.3389/fcimb.2016.00148

**Published:** 2016-11-10

**Authors:** Tatiana E. Erova, Michelle L. Kirtley, Eric C. Fitts, Duraisamy Ponnusamy, Wallace B. Baze, Jourdan A. Andersson, Yingzi Cong, Bethany L. Tiner, Jian Sha, Ashok K. Chopra

**Affiliations:** ^1^Department of Microbiology and Immunology, University of Texas Medical BranchGalveston, TX, USA; ^2^Department of Veterinary Sciences, University of Texas M. D. Anderson Cancer CenterBastrop, TX, USA; ^3^Institute for Human Infections and Immunity, University of Texas Medical BranchGalveston, TX, USA; ^4^Sealy Center for Vaccine Development and World Health Organisation Collaborating Center for Vaccine Research, University of Texas Medical BranchGalveston, TX, USA; ^5^Center for Biodefense and Emerging Infectious Diseases, University of Texas Medical BranchGalveston, TX, USA

**Keywords:** *Salmonella enterica* serovar Typhimurium, mouse model of salmonellosis, braun or murein lipoprotein (Lpp), lipopolysaccharide (LPS), acetyltransferase (MsbB), innate and adaptive immune responses, oral live-attenuated vaccine, 2-dimensional gel electrophoresis and analysis

## Abstract

We evaluated the extent of attenuation and immunogenicity of the Δ*lppAB* and Δ*lppAB* Δ*msbB* mutants of *Salmonella enterica* serovar Typhimurium when delivered to mice by the oral route. These mutants were deleted either for the Braun lipoprotein genes (*lppA* and *lppB*) or in combination with the *msbB* gene, which encodes an acetyltransferase required for lipid A modification of lipopolysaccharide. Both the mutants were attenuated (100% animal survival) and triggered robust innate and adaptive immune responses. Comparable levels of IgG and its isotypes were produced in mice infected with wild-type (WT) *S. typhimurium* or its aforementioned mutant strains. The Δ*lppAB* Δ*msbB* mutant-immunized animals resulted in the production of higher levels of fecal IgA and serum cytokines during later stages of vaccination (adaptive response). A significant production of interleukin-6 from T-cells was also noted in the Δ*lppAB* Δ*msbB* mutant-immunized mice when compared to that of the Δ*lppAB* mutant. On the other hand, IL-17A production was significantly more in the serum of Δ*lppAB* mutant-immunized mice (innate response) with a stronger splenic T-cell proliferative and tumor-necrosis factor-α production. Based on 2-dimensional gel analysis, alterations in the levels of several proteins were observed in both the mutant strains when compared to that in WT *S. typhimurium* and could be associated with the higher immunogenicity of the mutants. Finally, both Δ*lppAB* and Δ*lppAB* Δ*msbB* mutants provided complete protection to immunized mice against a lethal oral challenge dose of WT *S. typhimurium*. Thus, these mutants may serve as excellent vaccine candidates and also provide a platform for delivering heterologous antigens.

## Introduction

*Salmonella enterica* serovar Typhimurium is a food-borne pathogen that causes self-limiting gastroenteritis in healthy individuals. The global burden for non-typhoidal salmonellosis (NTS) was estimated at 93 million cases and 155,000 deaths in 2010 (Majowicz et al., 2010). Infection with *S. typhimurium* in immunologically compromised adults (e.g., HIV^+^) and children under the age of three, may lead to invasive non-typhoidal salmonellosis (iNTS) characterized by systemic infection and bacteremia, particularly in Africa and parts of south-east Asia, with approximately one million clinical cases per year (Feasey et al., 2012). The case fatality rate for iNTS bacteremia was reported at 25% (Reddy et al., 2010; Gordon, 2011). Antibiotics are the first choice to treat *Salmonella* infections; however, the rapidly emerging antibiotic resistance among *Salmonella* serovars has been a significant concern (Anderson, 1975). In the United States, it is estimated that ~7% of NTS infections are invasive, of which about 5% are fatal (Vugia et al., 2004). NTS strains resistant to multiple antibiotics complicate the treatment of iNTS (Anderson, 1975; Varma et al., 2005). In addition, *Salmonella* can be used as a bioweapon, as occurred in the intentional contamination of restaurant salad bars in Oregon by a religious cult, which resulted in ~1000 cases of gastroenteritis (Greenfield et al., 2002).

Currently, there is no vaccine available for NTS in humans. Several *S. typhimurium* mutants such as Δ*aroA* (Hormaeche et al., 1990, 1991), Δ*crp* Δ*cdt* (Zhang X. et al., 1997; Zhang et al., 1999), Δ*phoP* (Galán and Curtiss, 1989), Δ*relA* Δ*spoT* (Na et al., 2006), or Δ*aroC* Δ*ssaV* (designated as WT05; Hindle et al., 2002) have been developed and showed attenuation in mice. These deleted genes have been implicated in a variety of biological functions. For example, the *aroA* and *aero*C are involved in the bacterial aromatic (Aro) pathway (Hormaeche et al., 1991), while the *cdt* gene product plays an important role in bacterial colonization of deep tissues in the host (Zhang X. et al., 1997; Zhang et al., 1999). Likewise, the *crp* gene encodes cyclic AMP receptor protein and acts as a global transcriptional regulator (Shimada et al., 2013). A similar regulatory role has also been assigned to PhoP (Groisman et al., 1989). On the other hand, RelA and SpoT are responsible for synthesizing bacterial signal molecule ppGpp (Pizarro-Cerdá and Tedin, 2004), while *ssaV* encodes a crucial inner membrane structure component of the type III secretion system (T3SS) on the pathogenicity island 2 (SPI-2) of *Salmonella* (Hindle et al., 2002).

Although, animals vaccinated with the aforementioned mutants were protected against a dose >10^4^-fold above the LD_50_ of the parental *Salmonella* strains, these mutants were found to be either reactogenic or had disappointing immunogenicity in human clinical trials (Tennant et al., 2011; Strugnell et al., 2014). In addition, these live attenuated *S. typhimurium* vaccine strains were shed in the feces for longer periods of time, which is an undesirable trait for any vaccine (Tennant et al., 2011).

To address the rising concerns of iNTS, several new gene targets (e.g., *guaBA* and *clpP* or *lon*, encoding guanine biosynthesis proteins and ATP-dependent protease, respectively) have been deleted singly or in combination with other genes from *S. typhimurium* and *S. enteritidis* (Tennant et al., 2011; Matsui et al., 2015). Importantly, the *lon* and *sulA* (encoding the suppressor of *lon*) double deletion mutants of *S. typhimurium* and *S. enteritidis* showed cross protection in animal models (Tennant et al., 2011; Matsui et al., 2015). In addition, attenuated *Salmonella* strains have been used as vehicles to deliver foreign antigens. For example, a novel attenuated *S. typhimurium* strain SL368 derived from the auxotrophic *S*. *typhimurium aroA* strain SL7207 by deleting part of the *spiR* coding sequence, was used to express hemagglutinin as well as neuraminidase of a highly pathogenic H5N1 influenza virus. This strain provided protection to mice against both H5N1 and H1N1 viral infections (Pei et al., 2015). Likewise, an oral vaccine for type 1 diabetes was based on live attenuated *S. typhimurium* strain Mvp728 (Δ*htrA* Δ*purD*) that expressed diabetic autoantigen preproinsulin and transforming growth factor (TGF)-β (Husseiny et al., 2014).

One of the most serious complications of *S. typhimurium* infection is septic shock in humans and animals, which is mainly mediated by lipopolysaccharide (LPS) (Parrillo, 1993). We previously reported that Braun (murein) lipoprotein (Lpp) also contributed significantly to septic shock induction (Sha et al., 2004; Fadl et al., 2005a,b). Lpp is 5- to 9- kDa in size (Braun and Hantke, 1974; Braun, 1975; Zhang H. et al., 1997; Fenton and Golenbock, 1998) and encoded by two functional copies of the *lpp* gene (*lppA* and *lppB*) which are located in tandem and separated by 82 bp on the chromosome of *S. typhimurium* 14028 (Sha et al., 2004). Lpp synergizes with LPS to produce pro-inflammatory cytokines/chemokines (Braun, 1975; Sha et al., 2004; Fadl et al., 2005b). While LPS activates cellular responses by binding to CD14 receptor and *via* Toll-like receptor (TLR)-4 (Ulevitch and Tobias, 1995; Aliprantis et al., 1999; Tobias et al., 1999), Lpp triggers TLR-2 to activate host cell signaling (Aliprantis et al., 1999). In *Escherichia coli* and *S. typhimurium*, the *msbB* (multi-copy suppressor of *htrB* [high temperature requirement B]) gene encodes an acyltransferase that catalyzes the addition of lauric acid (C_12_) to the lipid A moiety of LPS, thus increasing its biological potency (Clementz et al., 1996, 1997; Somerville et al., 1996; Rebeil et al., 2006). Mutation in the *msbB* gene impaired *Salmonella*'s ability to cause lethality in mice and to induce tumor necrosis factor (TNF)-α, interleukin (IL)-1β, and inducible nitric oxide (iNOS) production (Kalupahana et al., 2003).

In our previous studies, we generated various individual (Δ*lppA* and Δ*lppB*) and combinatorial *lpp* and *msbB* (Δ*lppAB*, Δ*lppA* Δ*msbB*, Δ*lppB* Δ*msbB*, and Δ*lppAB* Δ*msbB*) gene deletion mutants of *S. typhimurium* 14028 and characterized them both *in vitro* and *in vivo* models of salmonellosis (Sha et al., 2004; Fadl et al., 2005a,b; Liu et al., 2008). We demonstrated that mice intraperitoneally (i.p.) immunized with these various mutant strains were protected from the lethal challenge dose (given by the i.p. route) of wild-type (WT) *S. typhimurium*. Furthermore, serum IgG1 antibody titers, T-cell proliferation, as well as the expression of T cell activation maker CD44, were substantially higher in mice i.p. immunized with the mutant strains when compared to that of WT *S. typhimurium*-infected animals (Liu et al., 2008). Both the Δ*lppAB* and Δ*lppAB* Δ*msbB* mutants were among the most attenuated and immunogenic ones, and, therefore, were considered as excellent vaccine candidates against *S. typhimurium* infection (Liu et al., 2008).

Oral inoculation is not only the natural route of *Salmonella* infection in the host but also the easiest and least invasive method of immunization. In the present study, we analyzed the immunological responses, including IgA levels, of inbred (C57BL/6J) mice that were orally immunized with the Δ*lppAB* or Δ*lppAB* Δ*msbB* mutants. In addition, we studied bacterial protein profiling alterations attributed to the *lpp* and/or *msbB* gene deletions by 2-dimensional (2D) gel electrophoresis and analysis. We detected changes in the levels of several potential immunogenic proteins in both the mutant strains, and demonstrated that Δ*lppAB* and Δ*lppAB* Δ*msbB* mutants induced robust innate and adaptive immune responses in mice and protected them from the lethal oral challenge with WT *S. typhimurium*. Our presented data further validate the vaccine potential of the mutants and we provide a possible mechanistic basis for the protection provided by these mutants in mice.

## Materials and methods

### Bacterial strains

WT *S. enterica* serovar Typhimurium 14028 was purchased from American Type Culture Collection (ATCC), Manassas, VA. The Δ*lppAB* and Δ*lppAB* Δ*msbB* mutants were generated in our laboratory (Sha et al., 2004; Fadl et al., 2005a,b). The *Salmonella* strains were grown either in Luria-Bertani (LB) or on MSB medium, the latter consisted of LB medium with no NaCl but supplemented with 2 mM MgSO_4_ and 2 mM CaCl_2_ (Murray et al., 2001). Bacterial cells from the exponential growth phase at 37°C were harvested and used for both animal studies (immunization and challenge) and 2D gel electrophoresis analysis. All of the *Salmonella* strains were periodically examined on *Salmonella-Shigella* (SS) agar plates (Difco, Detroit, MI) for purity.

### Serum antibodies and cytokines as well as fecal IgA in mice orally infected with *S. typhimurium* strains

Six-to-eight week old C57BL/6J female mice were obtained from the Jackson Laboratory (Bar Harbor, ME). Animals were first fasted for at least 4 h and then orally dosed with 100 μl of bacterial suspension with a mouse stomach tube (Sigma, St. Lois, MO). The bacterial doses were prepared in 5% sodium bicarbonate solution to overcome stomach acidity. Briefly, mice (*n* = 10 per group) were orally infected with 2.0 × 10^6^ colony forming units (CFU)/100 μl of WT *S. typhimurium* (our calculated LD_50_ was 2.0 × 10^5^ CFU) or its Δ*lppAB* or Δ*lppAB* Δ*msbB* mutants. Blood from the surviving animals from four independent experiments was collected by retro-orbital bleeding on days 0, 1, 3, 7, 14, and 21 post infection (p.i.). Total IgG and its isotype antibody titers to the whole bacterial cells on days 14 and 21 p.i. were evaluated by ELISA in serially diluted serum as we previously described (van Lier et al., 2014). Briefly, ELISA plates were first coated with the whole bacterial cells overnight at 4°C. For preparing the bacterial cells, the WT *S. typhimurium* was grown at 37°C until an OD_600nm_ of 0.8 was reached. The culture was then resuspended to a concentration of 5 × 10^9^ CFU/ml and used to coat the plates treated with poly-L-lysine (10 μg/ml). A serial dilution (1:5) of serum was made to evaluate antibody titers, and a positive antibody titer was defined as the inverse of the highest serum dilution giving an absorbance reading of ≥0.2. Antibody classes and IgG isotypes were also examined by using specific isotype secondary antibodies as we previously described (van Lier et al., 2014).

Likewise, the serum cytokine levels were examined on days 1, 3, 7, 14, and 21 p.i. by using a mouse 6-plex assay kit (Bio-Rad Laboratories Inc., Hercules, CA; Tiner et al., 2015). The fecal matter was collected on day 21 p.i. and suspended in phosphate-buffered saline (PBS) containing 0.15 mg soybean trypsin inhibitor and 25 mM EDTA (ethylenedinitrilo tetraacetic acid) (Sigma-Aldrich, St. Louis, MO). To measure the level of IgA, the ELISA plates were first coated with anti-IgA antibody (without biotinylation), and then the serial dilutions of fecal suspension were added to the plates. After 2 h incubation at room temperature followed by serial washes, the biotinylated anti-IgA antibody with the peroxidase-labeled streptavidin were applied. After further incubation, washes, and addition of the substrate, the plates were read under OD_450mm_ and the values were normalized by their corresponding total protein concentrations (μg/ml) in the fecal suspensions (Cong et al., 1998; Cao et al., 2012). Four independent experiments were performed and the pooled data (each sample was run in duplicate) were presented with statistical analysis. All of the animal studies were performed under an approved Institutional Animal Care and Use Committee protocol.

### Oral immunization, challenge, and histopathological analysis

C57BL/6J mice (*n* = 10 per group) were orally immunized/infected with 3 × 10^3^ CFU/100 μl of WT *S. typhimurium* and its Δ*lppAB* or Δ*lppAB* Δ*msbB* mutants. After 36 days, mice were challenged *via* the oral route with 2 × 10^6^ CFU/100 μl of WT *S. typhimurium*. Our goal was to discern efficacy of the vaccine strains at a low dose of 3 × 10^3^ CFU.

In a parallel experiment, mice (*n* = 5 per group) were orally immunized with a high dose of 1 × 10^8^ CFU (~1000 LD_50_ of WT *S. typhimurium*) of Δ*lppAB* or the Δ*lppAB* Δ*msbB* mutant and all survived. After 21 days, the immunized animals along with naïve control mice (*n* = 5) were challenged with 1 × 10^8^ CFU of WT *S*. *typhimurium via* the oral route. Organs from these challenged animals were excised on either day 7 (for unimmunized mice that were infected with WT *S. typhimurium* before succumbing to infection) or day 21 (for immunized and challenged animals, all survived). The organs were fixed in 10% neutral buffered formalin (Sha et al., 2008; Agar et al., 2009) and tissues processed and sectioned at 5 μm. The samples were mounted on slides and stained with hematoxylin and eosin (H&E). Tissue lesions were scored on the basis of a severity scale, which correlated with estimates of lesion distribution and the extent of tissue involvement, as we previously described (Sha et al., 2008; Agar et al., 2009). The histopathological evaluation of the tissue sections was performed in a blinded fashion.

### T-cell proliferative responses and cytokine production

Mice (*n* = 5) were orally immunized with 1 × 10^8^ CFU of Δ*lppAB* or the Δ*lppAB* Δ*msbB* mutant, and spleens were harvested on day 21 post immunization. We chose a higher dosage of the mutants for vaccination as *Salmonella* vaccines are administered at a dosage range of 10^7^-10^10^ CFU in humans, e.g., *S*. *typhi* Ty21a (WHO, 2014) or other typhoid vaccine candidates that have undergone clinical trials (Tacket et al., 2000; Tacket and Levine, 2007). T-cells from the mutant-immunized mice were isolated, and their ability to proliferate as well as to produce cytokines was evaluated upon co-culture with the γ-irradiated and heat-killed WT *S. typhimurium*-stimulated antigen-presenting cells (APCs; pulsed). T-cells incubated with γ-irradiated naïve APCs (without the bacterial stimulation; un-pulsed) served as controls (Sha et al., 2013; van Lier et al., 2014). After 72 h of incubation, 1μCi of [^3^H] thymidine was added to a set of each co-culture well, and the cells harvested 16 h later using a semi-automated sample harvester, FilterMate Harvester (PerkinElmer, Waltham, MA), followed by the measurement of radioactive counts (TopCount NXT, PerkinElmer). Likewise, from another set of the T-cell cultures, a portion of the supernatant was collected at 48 and 72 h to measure cytokine/chemokine production by using a mouse 6-plex assay kit (Bio-Rad Laboratories Inc.).

### Two-dimensional (2D) gel electrophoresis and mass spectrometric analysis

Bacterial cells (mutants and WT *S. typhimurium*) were lysed in 8 M urea, 4% CHAPS [3-(3-cholamidopropyl)-dimethylammonio-1-propanesulfonate], and 40 mM Tris-HCl (pH 8.0), followed by 10% (v/v) trichloroacetic acid (TCA) precipitation to remove salts and to concentrate proteins. The TCA precipitated proteins were re-dissolved in the lysis solution and subjected to 2D gel electrophoresis as we previously described (Chopra et al., 2006).

For each vaccine strain, the above prepared samples were run in triplicate gels, and the WT *S. typhimurium* sample-containing gels were set as a reference during analysis by using Progenesis Workstation (Nonlinear_Dynamics_, Durham, NC) at the Protein Chemistry Laboratory, UTMB. The normalized volume (NV) of each spot was calculated by using the total volume normalization method in which each spot on a gel image was expressed relative to the total volume of all spots on that image, and then normalized to the total volume of all spots on the reference gel image (Berth et al., 2007). The detected spots with NVs of ≤60 were filtered out. The NVs of the corresponding spots from gels containing samples of WT *S. typhimurium* and its two mutants were compared, and a fold-change of ≥2 was considered as differentially expressed/produced proteins. Some of these well separated protein spots (a total of 61) were picked robotically, trypsin-digested, and peptides identified by Matrix-Assisted Laser Desorption Ionization Time-of-Flight Mass Spectrometry (MALDI TOF-MS) at the Protein Chemistry Laboratory, UTMB.

### Statistics

Two-way analysis of variance (ANOVA) with the Tukey's *post-hoc* test or the multiple Student's *t*-test with the Holm-sidak *post-hoc* test correction was used for data analysis. We used Kaplan–Meier survival estimates or Fisher exact test for animal studies, and *p* ≤ 0.05 were considered significant for all of the statistical tests used.

## Results

### Attenuation of the Δ*lppAB* and Δ*lppAB* Δ*msbB* mutants of *S. typhimurium* in an oral mouse model of infection

**Figure 1:**
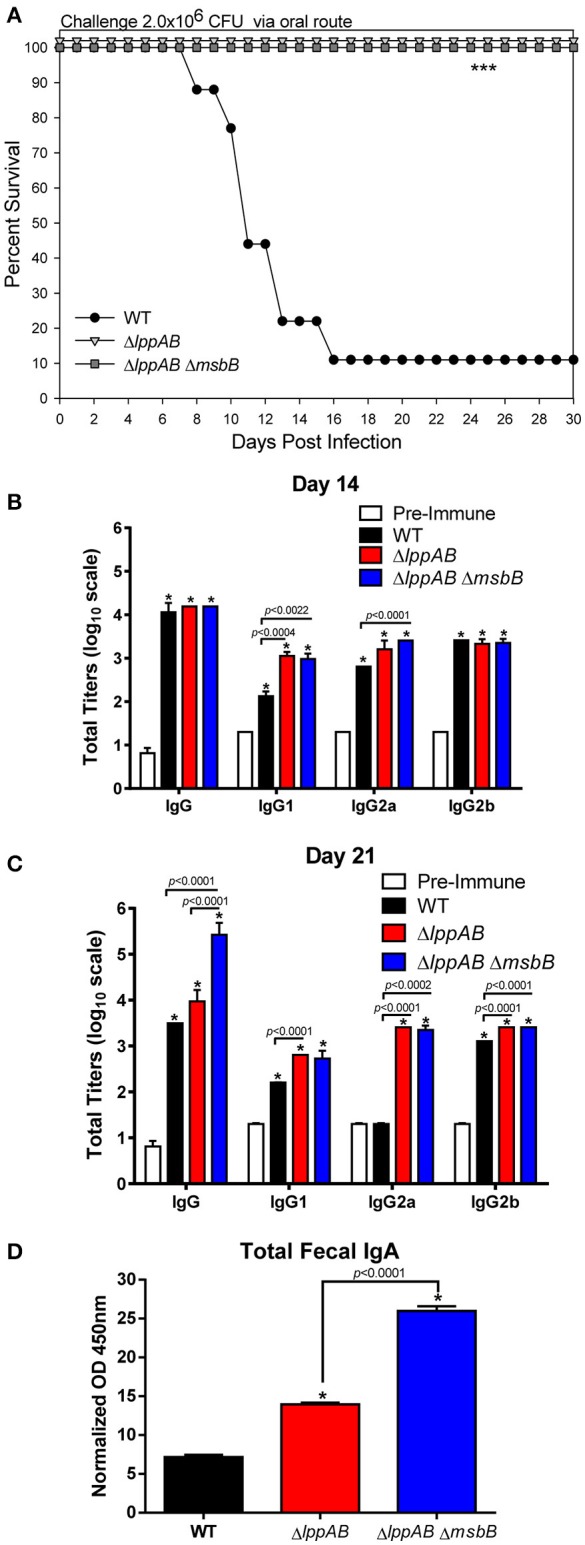
**Survival analysis, serum antibodies levels, and fecal IgA production in mice orally infected with WT and mutants of ***S. typhimurium*****. Six-to-eight week old C57BL/6J female mice (10 per group) were challenged with 2.0 × 10^6^ CFU/100 μl of WT *S. typhimurium* or its Δ*lppAB* and Δ*lppAB* Δ*msbB* mutants *via* the oral route. Mice were monitored for clinical scores, and the percentage survival in each group was plotted **(A)**. Asterisks indicate statistically significant *P*-value in comparison to the WT *S. typhimurium*-infected group and was based on Kaplan–Meier Curve Analysis (0.0027) and Fisher exact test (0.0079). Blood from animals was collected and the titers of IgG and its isotypes (serial dilutions of serum) to the whole bacterial cells on days 14 **(B)** and 21 **(C)** post infection (p.i.) were evaluated by ELISA. Serum collected on day 0 before infection served as a pre-immune control. Data were analyzed by using multiple Student's *t*-test with the Holm–sidak *post-hoc* test correction. The statistical significances were indicated either by asterisks when comparted to the pre-immune serum or by a line for the compared groups with the *P*-values. Mice fecal matter was collected on day 21 p.i., and the level of IgA in the fecal suspension was assessed by ELISA and the OD_450mm_ values were normalized by their corresponding total protein concentrations (μg/ml) in the fecal suspensions **(D)**. Two-way ANOVA with the Tukey's *post-hoc* correction was used to analyze the data, and the statistical significances were indicated either by asterisks when compared to WT *S*. *typhimurium*-infected mice or by a line for the compared groups with the *P*-value. The presented data were pooled from 4 independent experiments representing 4–8 animals/group.

Our previous studies with both Δ*lppAB* and Δ*lppAB* Δ*msbB* mutants in mice were focused on the i.p. model of infection (Sha et al., 2004; Fadl et al., 2005a,b; Liu et al., 2008). Both the mutant strains displayed significantly decreased virulence phenotype and provided protection to mice upon subsequent WT *S. typhimurium* lethal challenge. However, their potential as vaccine strains (attenuation and protection) in the oral infection mouse model was not characterized and formed the basis of this study. As shown in Figure 1A, when mice were orally challenged with WT *S. typhimurium* at the dose of 2.0 × 10^6^ CFU (~10LD_50_), animals started to die at day 8 p.i., and 90% of them eventually succumbed to infection by day 16 p.i. In sharp contrast, 100% of the mice infected with either the Δ*lppAB* and or the Δ*lppAB* Δ*msbB* mutant at the same dose of 2.0 × 10^6^ CFU survived up to tested 30 days, and no signs of the disease were observed during the course of infection (Figure 1A).

### Mouse serum antibody titers and fecal IgA production

Specific anti-*S. typhimurium* antibodies were evaluated on days 14 and 21 p.i. from the above infected groups of mice. Compared to the pre-immune serum, animals in all of the infected groups (WT *S. typhimurium* and the Δ*lppAB* and Δ*lppAB* Δ*msbB* mutants) developed high levels (~1:10,000) of *Salmonella* specific IgG antibodies on day 14 p.i. (Figure 1B). More specifically, all infected mice had similar levels of total IgG and IgG2b isotypes; however, slightly higher levels of IgG1 and IgG2a were observed in the mutant-infected groups of animals when compared to mice in the WT *S. typhimurium*-infected group (Figure 1B).

On day 21 p.i., the total IgG titers remained unchanged in the Δ*lppAB* mutant-infected mice, while it slightly dropped in animals infected with WT *S. typhimurium* when compared to that on day 14 p.i. (Figures 1B,C). In contrast, the total IgG titers in the Δ*lppAB* Δ*msbB* mutant-infected mice continued to mount (>1:100,000) on day 21 p.i. (Figure 1C). Likewise, the IgG isotype titers in all of the infected mice were maintained essentially at the similar levels when comparisons were made for days 14 and 21 p.i., with the exception of the level of IgG2a in WT *S. typhimurium*-infected animals which dropped back to the level detected in the pre-immune serum (Figure 1C).

The intestinal secretory IgA was also monitored during the course of infection, and a significant level of fecal IgA was detected on day 21 p.i. from all of the infected mice, whether with WT *S. typhimurium* or its Δ*lppAB* or Δ*lppAB* Δ*msbB* mutants. However, the highest level of IgA was noted in mice infected with the Δ*lppAB* Δ*msbB* mutant followed by the Δ*lppAB* mutant, and then the WT *S. typhimurium*-infected group of animals (Figure 1D).

### Levels of cytokines in mice serum during the course of infection with WT *S. typhimurium* and its mutants

**Figure 2:**
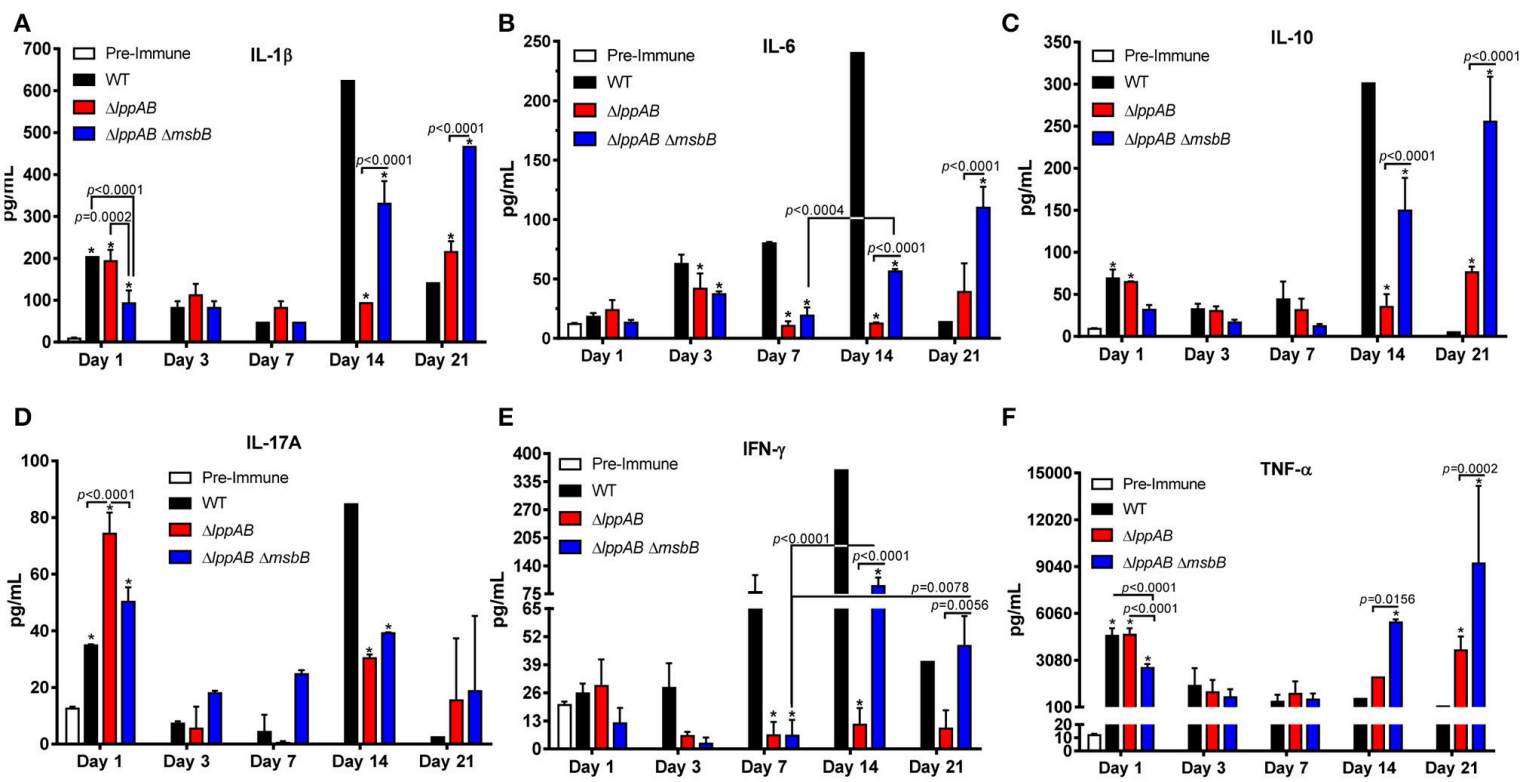
**Serum cytokine production in mice orally infected with WT and mutants of ***S. typhimurium*****. Six-to-eight week old C57BL/6J female mice (10 per group) were challenged with 2.0 × 10^6^ CFU/100 μl of WT *S. typhimurium* or its Δ*lppAB* and Δ*lppAB* Δ*msbB* mutants *via* the oral route. The serum cytokine levels IL-1β **(A)**, IL-6 **(B)**, IL-10 **(C)**, IL-17A **(D)**, IFN-γ **(E)**, and TNF-α **(F)** were examined on days 1, 3, 7, 14, and 21 p.i. by using a mouse 6-plex assay kit. Data were analyzed by using Two-way ANOVA with the Tukey's *post-hoc* correction. The statistical significances were indicated by a line for the compared groups with the *P*-values or by asterisks when compared to either the pre-immune serum on day 1 or the serum from WT *S. typhimurium*-infected mice for other days. The presented data were pooled from 4 independent experiments representing 4–8 animals/group.

Serum cytokines were monitored periodically by Bioplex during the course of infection. In general, irrespective of whether the mice were infected with WT *S. typhimurium* or its designated mutants, the levels of examined cytokines, except for IL-6 and interferon (IFN)-γ, were higher in the serum on day 1 p.i. when compared to the pre-immune serum (Figure 2). The levels of all examined cytokines were also maintained at significantly higher levels during the later stages of infection (i.e., days 14 or 21 p.i.), indicating the mounting of both early (innate) and late (adaptive) immune responses in the infected mice (Figure 2).

Interestingly, a similar trend in IL-1β, IL-10, IL-17A, and TNF-α production was observed in all of the infected groups of mice (i.e., WT *S. typhimurium* or its Δ*lppAB* or Δ*lppAB* Δ*msbB* mutants), in which their levels were relatively high on day 1 p.i., but subsequently declined by the end of the first week. However, a resurgence in these cytokine production reaching to their highest levels was noted on either day 14 or 21 p.i. (Figures 2A,C,D,F). On the other hand, the trends for IL-6 and IFN-γ production varied among different groups of mice (Figures 2B,E).

IFN-γ levels gradually mounted during the course of infection in the WT *S. typhimurium*-infected group of mice and reached the peak level on day 14 p.i. (Figure 2E). In contrast, a substantial level of IFN-γ was only detected on days 14 and 21 p.i. in the Δ*lppAB* Δ*msbB* mutant-infected group of mice. Strikingly, minimal IFN-γ was produced in the group of animals infected with the Δ*lppAB* mutant throughout the infection course (Figure 2E).

Likewise, the level of IL-6 gradually increased during the course of infection in the WT *S. typhimurium*-infected group of mice and reached the highest level on day 14 p.i. However, the levels of IL-6 in either of the mutant-infected mice increased somewhat on day 3 p.i. and declined on day 7 p.i., followed by its increase on days 14 or 21 p.i. (Figure 2B).

When comparing WT *S. typhimurium* vs. the mutant-infected mice, the former generally had higher levels of IL-1β, IL-6, IL-10, and IFN-γ on day 14 p.i. (Figures 2A–C,E). These cytokine levels significantly dropped to lower than that of both the mutant-infected groups of mice on day 21 p.i. On the other hand, TNF-α was maintained at generally a similar level across all of the infected mice (with WT *S. typhimurium* or its Δ*lppAB* or Δ*lppAB* Δ*msbB* mutants), but were elevated to much higher levels in both of the mutant-infected groups of animals at the later stages of infection (days 14 and 21, Figure 2F).

Interestingly, the production of IL-17A was generally higher in the mutant-infected groups of mice; however, the trend was reversed on day 14 p.i., with significantly higher levels of IL-17A in the WT *S. typhimurium* -infected group of animals (Figure 2D) compared to that of the mutant-infected groups of mice.

Finally, all of the measured cytokine levels were at the comparable levels in the Δ*lppAB* mutant-infected group of mice during the first week of infection in comparison to the Δ*lppAB* Δ*msbB* mutant-infected group of animals. However, the trend changed at the later stages of infection (e.g., days 14 and 21 p.i.), during which time significantly higher levels of cytokines were observed in the Δ*lppAB* Δ*msbB* mutant-infected group of mice (Figure 2).

### Protection conferred by oral immunization of mice with the Δ*lppAB* or the Δ*lppAB* Δ*msbB* mutant against subsequent WT. *S. typhimurium* challenge

**Figure 3:**
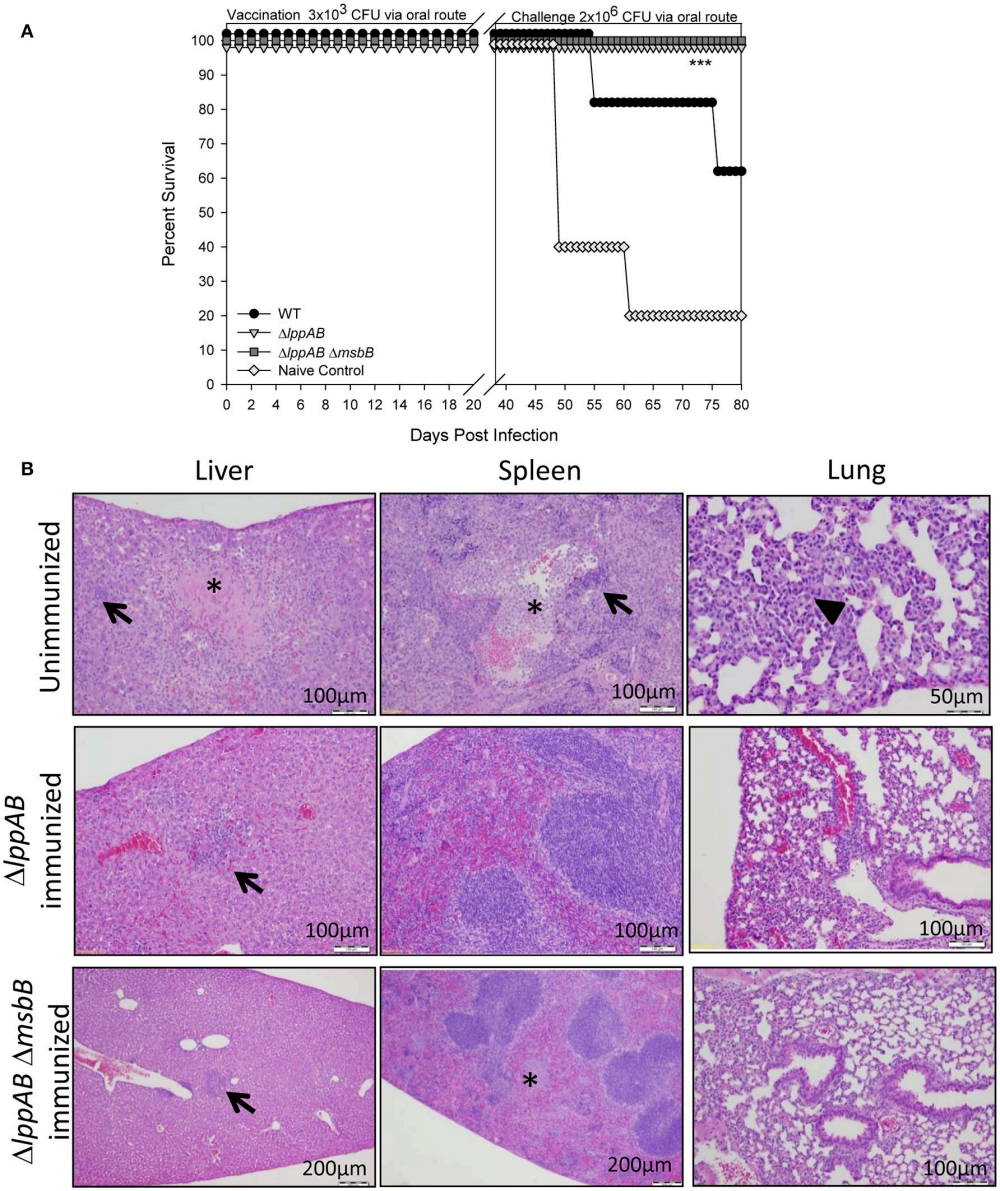
**Protection of mice conferred by immunization with the Δ***lppAB*** and Δ***lppAB*** Δ***msbB*** mutants of ***S. Typhimurium*** and histopathological analysis. (A)** Six-to-eight week old C57BL/6J female mice (10 per group) were infected with 3 × 10^3^ CFU/100 μl of WT *S. typhimurium* or its Δ*lppAB* and Δ*lppAB* Δ*msbB* mutants *via* the oral route. Naïve mice received PBS and served as a negative control. Thirty-six days after immunization, mice were orally challenged with 2 × 10^6^ CFU/100 μl WT *S. typhimurium*. Mice were monitored during immunization and subsequent challenge for clinical scores. The percentage survival of mice in each group was plotted. Asterisks indicate statistically significant *P*-value in comparison to the naïve control group and were based on Kaplan–Meier Curve Analysis (0.013) and Fisher exact test (0.04). **(B)** Mice (*n* = 5 per group) were orally immunized with 1 × 10^8^ CFU of Δ*lppAB* or the Δ*lppAB* Δ*msbB* mutant, and after 21 days, the immunized animals along with unimmunized naïve control mice (*n* = 5) were challenged with 1 × 10^8^ CFU of WT *S*. *typhimurium via* the oral route. Organs were excised from WT *S. typhimurium*-challenged unimmunized control mice on day 7 and from the mutant-immunized animals on day 21 post challenge with WT *S. typhimurium*. These organs were stained with H&E and evaluated by light microscopy in a blinded fashion. Asterisks indicate focal necrosis and thrombosis. Solid triangle indicates leukocytic infiltrate, while arrows indicate granulomas. Magnification of the images were also shown. Different magnifications chosen for various panels were to show either detailed histopathologic alterations or a normal tissue architecture over a broader area.

To gauge protection provided by immunization of mice with the mutant strains compared to that of the WT bacterium, animals were initially immunized/infected orally with a relatively low dose (3 × 10^3^ CFU) of the mutant strains (Δ*lppAB* and Δ*lppAB* Δ*msbB*) or WT *S. typhimurium*. This low dose ensured animal survival from WT *S. typhimurium* infection as its LD_50_ was calculated to be 2.0 × 10^5^ CFU when administered *via* the oral route. The naïve mice received PBS and served as a control. No animal succumbed to infection during the initial immunization/infection period in all of the groups. On day 36 post immunization, these mice were orally challenged with 2 × 10^6^ CFU of WT *S. typhimurium* (Figure 3A). All mice vaccinated with either the Δ*lppAB* or the Δ*lppAB* Δ*msbB* mutant survived, while 60% of the animals were protected in the WT *S. typhimurium*-vaccinated group. Naïve control mice (80%), on the other hand, succumbed to infection.

In a parallel experiment, mice were orally immunized with a high dose (1 × 10^8^ CFU) of Δ*lppAB* or the Δ*lppAB* Δ*msbB* mutant, and no animal succumbed to infection during immunization. After 21 days, the immunized mice were challenged with WT *S. typhimurium* (1 × 10^8^ CFU) *via* the oral route and all survived. Mouse organs were excised 21 days post challenge for histopathological analysis. The high doses used for both immunization and challenge were to examine safety of the aforementioned mutants and their protective potential. The unimmunized naïve mice challenged with 1 × 10^8^ CFU of WT *S. typhimurium via* the oral route were used as a positive control for examining histopathological lesions in organs. These tissues were examined on day 7 post challenge as animals showed severe symptoms such as ruffled fur, lethargy, loss in weight, and diarrhea. As shown in Figure 3B, the WT *S. typhimurium*-challenged naïve mice had pyogranulomas in various peripheral organs, such as the liver and spleen (arrows), and the lungs had inflammatory cell infiltrate (solid triangle). The splenic tissues revealed lymphoid depletion in white pulps and leukocytosis. The depleted cell types were more likely hematopoietic or stromal cells in the red pulps. In addition, focal necrosis and thrombosis were observed in liver and spleen tissues (asterisks) of mice infected with the WT bacterium. However, intestinal tissue sections, e.g., duodenum, jejunum, and ileum, showed no marked lesions except for inflammation on day 7 p.i. (data not shown).

While analyzing tissues from both of the mutant-immunized groups of mice after WT *S. typhimurium* challenge, the above observed lesions (e.g., granulomas in the liver, arrows) and focal necrosis/thrombosis (asterisk) in the spleens were much milder in nature with largely intact red and white pulps, when compared to tissues from the WT *S. typhimurium*-infected naïve mice (Figure 3B).

### T-cell responses in mice immunized with the Δ*lppAB* or the Δ*lppAB* Δ*msbB* mutant of *S. typhimurium*

**Figure 4:**
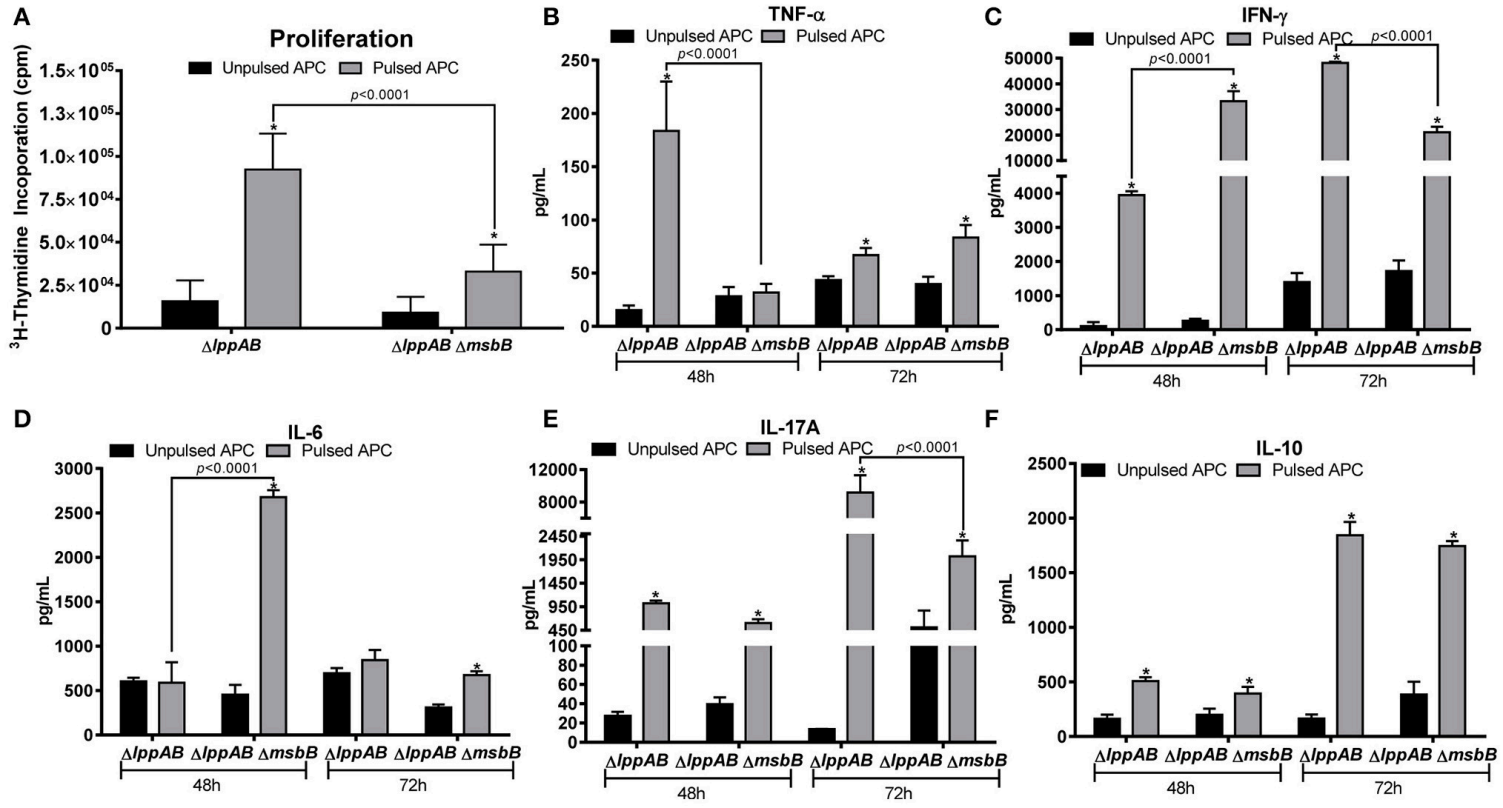
**T-cell proliferation in mice immunized with the Δ***lppAB*** and Δ***lppAB*** Δ***msbB*** mutants of ***S. typhimurium*** and cytokine production. (A)** Six-to-eight week old C57BL/6J female mice (*n* = 5) were orally immunized with 1 × 10^8^ CFU/100 μl of Δ*lppAB* or the Δ*lppAB* Δ*msbB* mutant, and spleens harvested at day 21 post immunization. T-cells were isolated and their ability to proliferate was evaluated upon co-culture with γ-irradiated and heat-killed WT *S. typhimurium* stimulated APCs (pulsed). T-cells incubated with γ-irradiated naïve APCs without the bacterial stimulation (un-pulsed) served as controls. Two-way ANOVA with the Tukey's *post-hoc* correction was used to analyze the data, and the statistical significances were indicated either by asterisks when compared to their un-pulsed controls or by a line for the compared groups with the *P*-values. After 48 and 72 h incubation, the culture supernatants from T-cells were harvested, and the production of cytokine TNF-α **(B)**, IFN-γ **(C)**, IL-6 **(D)**, IL-17A **(E)**, and IL-10 **(F)** were examined by using a mouse 6-plex assay kit. Two-way ANOVA with the Tukey's *post-hoc* correction was used to analyze the data, and the statistical significances were indicated either by asterisks when compared to their un-pulsed controls or by a line for the compared groups with *P*-values.

As shown in Figure 4A, T-cells from both the mutant-immunized groups of mice significantly proliferated in response to the stimulation with WT *S. typhimurium* antigens. However, the extent of proliferation was much higher for the Δ*lppAB* mutant-vaccinated mice than that of Δ*lppAB* Δ*msbB* mutant-immunized animals.

A robust cytokine production was also observed in T-cells from both the Δ*lppAB* and Δ*lppAB* Δ*msbB* mutant-immunized mice in response to stimulation with WT *S. typhimurium* antigens. At 48 h after stimulation, T-cell production of TNF-α in the Δ*lppAB*-immunized mice was significantly higher when compared to T-cells from the Δ*lppAB* Δ*msbB* mutant-immunized animals (Figure 4B). On the other hand, IFN-γ and IL-6 production from T-cells in the Δ*lppAB* Δ*msbB* mutant-immunized animals (Figures 4C,D) were much higher when compared to the animals immunized with the Δ*lppAB* mutant.

At 72 h after stimulation, T-cell production of IFN-γ (Figure 4C) and IL-17A (Figure 4E) were much higher in the Δ*lppAB* mutant-immunized mice when compared to that in the Δ*lppAB* Δ*msbB* mutant-vaccinated animals. The level of IL-10 was maintained at a similar level in both the mutant- immunized mice at both time points of 48 and 72 h (Figure 4F).

Compared to the un-pulsed controls, IFN-γ and IL-10 production were at higher levels in T-cells from both of the mutant infected mice (Figures 4C,F) at both the time points; however, IL-6 levels were specifically induced in T-cells of Δ*lppAB* Δ*msbB* mutant-immunized mice at 48 h time point (Figure 4D), Likewise, TNF-α was significantly produced only from the T-cells of Δ*lppAB* mutant-immunized mice upon stimulation with the WT *S. typhimurium* antigens at a 48 h time point, but it was significantly dropped at 72 h (Figure 4B). In contrast, the level of TNF-α in the supernatant of pulsed T-cells from the Δ*lppAB* Δ*msbB* mutant-immunized mice was slightly increased at 72 h time point and reached statistical significance when compared to the un-pulsed control (Figure 4B).

### Alterations in the protein profiling of Δ*lppAB* and Δ*lppAB* Δ*msbB* mutants in comparison to WT *S. typhimurium*

2D gel electrophoresis and analysis were performed to obtain protein profiling of WT *S. typhimurium* and that of its Δ*lppAB* and Δ*lppAB* Δ*msbB* mutants. There were a total of 1726, 1899, and 1835 protein spots on the 2D gels for WT bacteria, and its Δ*lppAB* and Δ*lppAB* Δ*msbB* mutants, respectively. After manual editing and filtering, a total of 1099 spots correlated across the gels of different groups of bacteria (e.g., WT *S. typhimurium*, Δ*lppAB*, and Δ*lppAB* Δ*msbB* mutants; Table S1). Compared to the 2D gels for WT *S. typhimurium*, 22 spots had higher normalized volumes (NVs) and 47 spots had lower NVs in the Δ*lppAB* mutant (Table S2). These numbers were 46 (higher) and 14 (lower) for the Δ*lppAB* Δ*msbB* mutant. Likewise, 10 spots on the 2D gel for the Δ*lppAB* mutant and 43 spots for the Δ*lppAB* Δ*msbB* mutant had relatively higher NVs when these two mutants were compared to each other (Table S2).

A total of 61 spots were picked based on their fold changes and NVs for mass spectrometric analysis. Among these identified proteins, the majority of them belonged to bacterial inner and outer membranes such as structural proteins, receptors, and transporters. In addition, some proteases, metabolic enzymes, translation associated components, as well as cell signaling related molecules (synthases and regulatory proteins) were also observed. Interestingly, the levels of two proteins, outer membrane protein A (OmpA) and periplasmic protein TolB were higher in both the Δ*lppAB* and Δ*lppAB* Δ*msbB* mutants when compared to that in the WT bacterium (Table 1).

**Table 1:** **Proteins altered in both Δ***lppAB*** and Δ***lppAB*** Δ***msbB*** mutants compared to WT ***S. typhimurium*** based on 2D gel electrophoresis and analysis**.

**Names of the proteins**	**Accession No**.	**Fold changes compared to WT^#^**
Outer membrane protein A	AAV77696	2.3 to 3.1
TolB	CAD05210	2.3 to 2.9
Serine protease	CAA38420	−3.6 to −3.8
Tricarboxylic transport protein	NP_461712	−2.2
cAMP-regulatory protein	YP_671327	−3.4 to −8.3
Arginine-binding periplasmic protein 2 precursor	CAD05326	−2.0 to −2.5
Flagellar-associated GTP-binding protein	AAU07126	−2.5 to −3.0
Esterase	WP_021000185	−2.5 to −4.1
Superoxide dismutase	AAV77363	−2.1 to −2.2

# *Based on the normalized volumes (NVs), and (−) sign denotes decreased production in the mutants*.

In contrast, the level of seven proteins, including a serine protease, tricarboxylic transport, cAMP-regulatory protein, arginine-binding periplasmic protein 2 precursor, flagellar-associated GTP-binding protein, esterase, and superoxide dismutase, were generally lower in both the Δ*lppAB* and Δ*lppAB* Δ*msbB* mutants (Table 1). Likewise, when comparison was made between the two mutants, DNA-directed RNA polymerase beta subunit (RpoB) and Rho factor were the two lead proteins produced significantly more with respective 8.1–39.4 and 7.4-fold changes (Table S3), respectively, in the Δ*lppAB* Δ*msbB* mutant. On the other hand, protein chain initiation factor 2 and Lon protease were increased by 7.0 and 4.4-folds, respectively, in the Δ*lppAB* mutant. A complete list of 61 spots with their protein identification and fold changes are shown in Table S3.

## Discussion

Vaccine-based prophylaxis has historically been not only the most significant advances in the healthcare, but also a cost-effective means of public health intervention. We generated *S. typhimurium* mutants deleted for the *lppA* and *lppB* genes alone or in combination with the *msbB* gene, and studied their attenuation and immunogenicity in mice first in a septicemic mouse model of infection (Sha et al., 2004; Fadl et al., 2005a,b; Liu et al., 2008), and now by the oral route.

Lpp synergizes with LPS to produce pro-inflammatory cytokines/chemokines by activating host cells through TLR-2 and TLR-4 signaling, respectively (Ulevitch and Tobias, 1995; Aliprantis et al., 1999; Tobias et al., 1999). In our previous studies, we showed that both Δ*lppAB* and Δ*lppAB* Δ*msbB* mutants indeed triggered much less pro-inflammatory cytokines compared to the WT bacterium in an intraperitoneal mouse model of infection during early stages of infection (within a week; Sha et al., 2004; Fadl et al., 2005a,b; Liu et al., 2008). However, this phenomenon was not apparent in mice orally infected with the mutants. For example, IL-17A was detected in higher levels in both the mutant-infected mice when compared to WT *S. typhimurium*-infected animals during early stages of infection (Figure 2). In addition, a more balanced Th1 and Th2 antibody responses were observed across all orally infected mice, irrespective of whether WT or mutants of *S. typhimurium* were used (Figures 1B,C). In contrast, IgG1 was the dominant isotype in all mice infected intraperitoneally, accompanied with high levels of T-cell IL-4 production from animals infected with the mutant strains (Liu et al., 2008). These differences may be attributed to different infection routes, bacterial dosages used, and, specifically, C57BL/6J mice are biased to mount a Th1 response (Watanabe et al., 2004).

IL-17 is a signature cytokine of Th17 cells, along with IL-21 and IL-22, and the Th17 response protects animals in an antibody-independent manner (Lin et al., 2011). It has been reported that IL-17 and its associated cytokines (IL-21 and IL-22) are induced in the gastrointestinal tract during *Salmonella* infection (Ramarathinam et al., 1993; Lee et al., 2012; Kurtz et al., 2014; McSorley, 2014). While Th1 cells are critical for activation of infected macrophages to kill *Salmonella* in the tissues, Th17 cells are likely essential for recruiting neutrophils to the site of intestinal infection to engulf bacteria (Ramarathinam et al., 1993; Tükel et al., 2009; Broz et al., 2012; Lee et al., 2012). Likewise, fecal IgA, the abundant class of antibodies in the intestinal secretion, serves as the first line of defense against infection (Michetti et al., 1992; Mantis et al., 2011; Gutzeit et al., 2014), and IgA antibodies against a variety of *Salmonella* antigens are highly effective in preventing salmonellosis (Michetti et al., 1992, 1994; Amarasinghe et al., 2013). Although both Th17 and IgA responses have been shown to provide protection against *Salmonella* infection (Martinoli et al., 2007; Ko et al., 2009; Mayuzumi et al., 2010; Geddes et al., 2011; Keestra et al., 2011), it is still unclear whether both the Δ*lppAB* and Δ*lppAB* Δ*msbB* mutant-induced Th17 and IgA responses in mice are directly linked to their protection against subsequent WT *S. typhimurium* challenge.

Nevertheless, the serum levels of IL-17A were not only generally maintained at higher levels in the mutant-infected mice when compared to WT bacterium-infected animals at an early infection stage (e.g., day 1), but were also sustained longer in the mutant-infected mice at the later infection stages (Figure 2D). More importantly, these IL-17A levels correlated with more fecal IgA production on day 21 p.i. in mice infected with the Δ*lppAB* and Δ*lppAB* Δ*msbB* mutants (Figure 1D). As we demonstrated recently that Th17 cell production of IL-17 promoted intestinal IgA responses (Cao et al., 2012), it is very likely that the increased IL-17 mediated the high fecal IgA response in mice immunized with the mutants.

In addition to higher IL-17A and fecal IgA production, increased levels of *Salmonella*- specific antibodies as well as other examined serum cytokines, which were generally sustained for longer periods (up to day 21 p.i.; Figures 1, 2) in mice orally infected with the mutants, might have contributed to full protection of immunized mice to WT bacterial challenge. This protection was 60% in WT *S. typhimurium* -infected mice during the subsequent re-challenge with WT bacterium (Figure 3A). Interestingly, each of the two mutants displayed some unique immunological aspects. For example, the Δ*lppAB* mutant-infected mice had relatively higher serum cytokines at the early infection stage (Figure 2), and elicited stronger T-cell proliferation which was associated with the production of TNF-α in comparison to animals infected with the Δ*lppAB* Δ*msbB* mutant (Figure 4).

On the other hand, the Δ*lppAB* Δ*msbB* mutant-infected mice generally displayed higher levels of serum cytokines during the later stages of infection (Figure 2) and IL-6 production from T-cells (Figure 4), as well as more fecal IgA production (Figure 1). However, it is unclear whether all of these differences are related to the compromised TLR-4 signaling as a result of the *msbB* deletion, which needs to be further investigated. Interestingly, LPS, a sero-dominant and protective antigen in most gram-negative bacteria, has undergone limited analysis as a human *Salmonella* vaccinogen (MacLennan et al., 2010). This is being re-addressed in new exploratory programs to develop *Salmonella* vaccines using LPS from *S*. *enteritidis* (Simon et al., 2011) and *S. typhimurium* (Simon et al., 2013) as part of efforts to combat iNTS (Strugnell et al., 2014). Most importantly, a vaccine using GMMA (generalized modules for membrane antigens) from *msbB* and *pagP* (encoding lipid A palmitoyltransferase) deletion mutants of *S. typhimurium* and *S. enteritidis* with reduced potential for *in vivo* reactogenicity showed a slightly higher stimulatory potential than WT *S. typhimurium* harboring WT lipid A (Rossi et al., 2016). Therefore, in this regard, the Δ*lppAB* Δ*msbB* mutant with modified LPS may have some advantages over the Δ*lppAB* mutant.

Based on 2D gel analysis (Table 1 and Table S3), the production of several proteins such as OmpA, OmpX, TolB, and superoxide dismutase were found to be altered in the Δ*lppAB* and/or Δ*lppAB* Δ*msbB* mutants compared to WT *S. typhimurium*. More specifically, the production of first three proteins was up-regulated while the production of superoxide dismutase was down-regulated in the mutants. OmpA binds to and activates APCs, resulting in protective cytotoxic T-lymphocyte (CTL) responses in *Klebsiella pneumoniae*. It also augments cytokine production (IL-1, -10, and -12) by dendritic cells (DCs) and their migration across the polarized human intestinal epithelial cells in *E. coli* O157:H7 (Jeannin et al., 2000; Maisnier-Patin et al., 2003; Torres et al., 2006). While OmpX, also referred to as Ail (attachment-invasion locus), is a major contributor to serum resistance and complement evasion in *Yersinia pestis* (Bartra et al., 2008; Tiner et al., 2015).

Importantly, active immunization with either OmpA or Ail (OmpX) provided protection to mice and rats against developing bubonic and/or pneumonic plague (Erova et al., 2013). The periplasmic protein TolB belongs to the gram-negative bacterial Tol system which comprises five proteins (TolQ, TolR, TolA, TolB, and Pal), and are involved in maintaining bacterial outer membrane stability (Lazzaroni et al., 2002). A study has shown that Groupers, e.g., *Epinephelus awoara*, a fish belonging to the family *Serranidae*, immunized with the recombinant TolB of *Vibrio alginolyticus* generated high antibody responses and protected the immunized fish from *V. alginolyticus* infection (Pang et al., 2015). The superoxide dismutase protects *Salmonella* from products of phagocyte NADPH-oxidase and nitric oxide synthase (De Groote et al., 1997). Consequently, the reduced production of superoxide dismutase in the Δ*lppAB* Δ*msbB* mutant compared to WT *S. typhimurium* might result in efficient killing of the mutant by phagocytic cells as we previously demonstrated (Fadl et al., 2005b), a feature desirable for any vaccine candidate (Golubeva and Slauch, 2006).

Interestingly, the level of flagellar-associated GTP-binding protein decreased in both Δ*lppAB* and Δ*lppAB* Δ*msbB* mutant strains based on our 2D gel analysis (Table 1), which was in agreement with the reduced transcription level of flagellar structure protein-encoding genes detected by our previous microarray analysis (Fadl et al., 2006), thus adding credibility to our data obtained by both 2D and microarray analyses. Flagellin, a TLR-5 agonist, has been recognized and used as an adjuvant for many vaccines (Hayashi et al., 2001; Yin et al., 2013; Gupta et al., 2014). However, a recent study showed that flagellin was not a major factor for GMMA-mediated immune stimulation against salmonellosis (Rossi et al., 2016). Therefore, the immunogenicity of our mutants either should not be significantly influenced by the decreased flagellar-associated proteins or can be further enhanced in conjunction with the flagellin based adjuvants.

In addition, a study with *Y. pestis* EV Δ*lpxM* (*msbB*) mutant also revealed pleiotropic effects of LpxM in altering synthesis of major immunoreactive antigens (Feodorova et al., 2009). Therefore, it will be interesting to further discern the role of the altered potential immunogens in triggering better immune responses by the mutants than the WT *S. typhimurium*, and explore the possibility of developing a subunit vaccine(s) against salmonellosis.

Nevertheless, the two attenuated *S. typhimurium* mutant strains, Δ*lppAB* and Δ*lppAB* Δ*msbB*, elicited robust innate and adapt immune responses in mice as measured by sera cytokines and specific *Salmonella* antibody levels, as well as T cell responses. Thus, both Δ*lppAB* and Δ*lppAB* Δ*msbB* mutants showed potential as viable vaccine candidates against *S. typhimurium* infection. In addition, the immunological differences observed between these mutants may provide unique perspective for specific applications. Our future direction will include studying the potential cross-protection conferred by the immunization of animals with Δ*lppAB* and Δ*lppAB* Δ*msbB* mutants of *S. typhimurium* against other *S. enterica* serovars (e.g., *S. enteritidis*).

## Author contributions

TE, MK, and JS planned as well as executed all of the experiments described above. EF and JA helped with animal experiments. DP performed the histopathological animal experiments and WB analyzed the histopathological data. MK, EF, and BT helped in data analysis and formatting the figures. YC and AC helped in the planning of all experiments and discussion of the acquired results. AC and JS also contributed to the writing and editing of the manuscript. AC is the guarantor.

### Conflict of interest statement

The authors declare that the research was conducted in the absence of any commercial or financial relationships that could be construed as a potential conflict of interest.
