# Supplementary material for: Protective Immunity Elicited by Oral Immunization of Mice with Salmonella enterica Serovar Typhimurium Braun Lipoprotein (Lpp) and Acetyltransferase (MsbB) Mutants
Source: Front Cell Infect Microbiol. 2016 Nov 10;6:148. doi: 10.3389/fcimb.2016.00148 (PMC5103298; doi:10.3389/fcimb.2016.00148)
Supplement: Supplementary file 1 [file Table1.DOCX]

| **Table S1. Number of spots detected on the 2D gels for WT *S*. Typhimurium and its mutants** | | |
| --- | --- | --- |
| Gel | After spot detection | After manual editing and filtering |
| WT (reference) | 1726 | 1099 |
| *∆lppAB* | 1899 | 1099 |
| ∆*lppAB* ∆*msbB* | 1835 | 1099 |
| Spots filtered by normalized volumes (NVs) of > 60 | | |

| **Table S2. Number of spots differentially expressed/produced on 2D gels for WT *S*. Typhimurium and its mutants** | | | |
| --- | --- | --- | --- |
|  | *∆lppAB* vs WT | ∆*lppAB* ∆*msbB* vs WT | *∆lppAB* vs ∆*lppAB* ∆*msbB* |
| Higher in WT | 47 | 14 | - |
| Higher in *∆lppAB* | 22 | - | 10 |
| Higher in ∆*lppAB* ∆*msbB* | - | 46 | 43 |
| Comparisons were based on the normalized volumes (NVs), and spots with ≥ 2 fold changes were listed. | | | |

| **Table S3. Protein identities of 2D gel spots with differential normalized volumes between WT, Δ*lppAB,* and Δ*lppAB* Δ*msbB* mutants of *S*. Typhimurium** | | | | |
| --- | --- | --- | --- | --- |
| **Spot Number** | **Protein Name** | **Source Organism*** | **Accession No.** | **Fold Change^#^** |
| **Higher in Δ*lppAB* vs WT** | | | | |
| 192 | Protein chain initiation factor 2 | *Salmonella enterica* subsp. enterica serovar Typhi str. CT18 | CAD07806 | 3.9 |
| 265 | Lon protease | *Salmonella enterica* subsp. enterica serovar Typhi str. CT18 | CAD08909 | 3.5 |
| 703 | Chain A, Crystal Structure of the E. Coli TolB protein | *Escherichia coli* | 1CRZ_A | 3.2 |
| 719 | TolB protein precursor | *Salmonella enterica* subsp. enterica serovar Typhi str. CT18 | CAD05210 | 2.9 |
| 891 | Outer membrane protein A | *Salmonella enterica* subsp. enterica serovar Paratyphi A str. ATCC 9150 | AAV77696 | 2.8 |
| 904 | Outer membrane protein A | *Salmonella enterica* subsp. enterica serovar Paratyphi A str. ATCC 9150 | AAV77696 | 2.5 |
| 979 | Outer membrane protein A | *Salmonella enterica* subsp. enterica serovar Paratyphi A str. ATCC 9150 | AAV77696 | 3.1 |
| 1108 | ABC transporter substrate-binding protein | *Salmonella enterica* | WP_000476475 | 2.4 |
| 1159 | Cyclic AMP receptor protein, catabolite gene activator | *Salmonella enterica* subsp. enterica serovar Typhi str. CT18 | CAD08147 | 2.2 |
| 1458 | Outer membrane protein X precursor; outer membrane protein X precursor | *Salmonella enterica* subsp. enterica serovar Typhi str. CT18 | CAD05280 | 2.2 |
| 1463 | Outer membrane protein X precursor; outer membrane protein X precursor | *Salmonella enterica* subsp. enterica serovar Typhi str. CT18 | CAD05280 | 2.2 |
| 1608 | γ-glutamylcyclotransferase | *Salmonella enterica* | WP_001219167 | 7.3 |
| **Higher in Δ*lppAB* Δ*msbB* vs WT** | | |  |  |
| 165 | DNA-directed RNA polymerase beta subunit (RpoB) | *Salmonella enterica* subsp. enterica serovar Typhimurium | AIE08112 | 2.3 |
| 188 | DNA-directed RNA polymerase beta subunit | *Salmonella enterica* subsp. enterica serovar Typhi str. Ty2 | NP_807130 | 8.5 |
| 256 | Iron-dependent alcohol dehydrogenase | *Salmonella enterica* subsp. enterica serovar Typhimurium str. LT2 | NP_460708 | 2.7 |
| 307 | Pyruvate dehydrogenase E1 component | *Salmonella enterica* subsp. enterica serovar Typhimurium str. LT2 | NP_459157 | 4.5 |
| 315 | Pyruvate dehydrogenase E1 component | *Salmonella enterica* subsp. enterica serovar Typhi str. CT18 | CAD01311 | 4.3 |
| 480 | Periplasmic glucans biosynthesis protein | *Salmonella enterica* subsp. enterica serovar Choleraesuis str. SC-B67 | YP_216084 | 2.3 |
| 493 | Periplasmic glucans biosynthesis protein | *Salmonella enterica* subsp. enterica serovar Choleraesuis str. SC-B67 | YP_216084 | 2.3 |
| 601 | Acetyl-CoA carboxylase | *Salmonella enterica* subsp. enterica serovar Choleraesuis str. SC-B67 | YP_218305 | 3.1 |
| 615 | Biotin carboxylase | *Salmonella enterica* subsp. enterica serovar Typhi str. CT18 | CAD07895 | 2.5 |
| 649 | AraC-type DNA-binding domain-containing protein | *Vibrio vulnificus* YJ016 | WP_011081127 | 2.9 |
| 703 | Chain A, Crystal Structure of the E. Coli TolB Protein | *Escherichia coli* | 1CRZ_A | 2.3 |
| 713 | TolB protein precursor | *Salmonella enterica* subsp. enterica serovar Typhi str. CT18 | CAD05210 | 2.5 |
| 726 | Glycerol-3-phosphate-binding periplasmic protein | *Salmonella enterica* subsp. enterica serovar Typhi str. CT18 | CAD08072 | 3.3 |
| 743 | D-alanyl-D-alanine-carboxypeptidase | *Salmonella enterica* subsp. enterica serovar Typhimurium str. LT2 | NP_459629 | 5.0 |
| 1158 | Outer membrane protein A | *Salmonella enterica* subsp. enterica serovar Paratyphi A str. ATCC 9150 | AAV77696 | 2.2 |
| 1365 | LPS transport periplasmic protein LptA | *Salmonella enterica* | WP_058818633 | 2.0 |
| 1402 | Molybdenum cofactor biosynthesis protein B | *Salmonella enterica* subsp. enterica serovar Typhi str. CT18 | CAD05251 | 3.1 |
| 1423 | Ribosomal protein S7 | *Salmonella enterica* subsp. enterica serovar Typhimurium | CAA45879 | 2.9 |
| 1577 | Ribosomal protein L19 | *Salmonella enterica* subsp. enterica serovar Typhimurium | CAA52888 | 2.5 |
| **Higher in WT vs Δ*lppAB*** | | | | |
| 400 | 30S ribosomal protein S1 | *Salmonella enterica* subsp. enterica serovar Typhi str. CT18 | CAD05381 | 2.7 |
| 461 | 2',3'-cyclic nucleotide 2'-phosphodiesterase | *Salmonella enterica* subsp. enterica serovar Paratyphi str. ATCC 9150 | AAV79958 | 2.7 |
| 481 | Chain N, Groel14-(atpgammas)14 | *Escherichia coli* | 1SX3_N | 2.0 |
| 541 | Phosphoenolpyruvate carboxykinase | *Salmonella enterica* subsp. enterica serovar Typhi str. CT18 | CAD08114 | 4.1 |
| 546 | Phosphoenolpyruvate carboxykinase | *Salmonella enterica* subsp. enterica serovar Typhi str. CT18 | CAD08114 | 2.6 |
| 594 | Rho factor | *Salmonella enterica* subsp. enterica serovar Typhimurium | CAA79852 | 4.7 |
| 818 | Isoaspartyl dipeptidase | *Salmonella enterica* subsp. enterica serovar Typhimurium str. LT2 | NP_463371 | 2.5 |
| 910 | Glyceraldehyde 3-phosphate dehydrogenase A | *Salmonella enterica* subsp. enterica serovar Typhi str. CT18 | CAD02064 | 4.5 |
| 929 | Sugar Specific PTS family, mannose-specific enzyme IIAB | *Salmonella enterica* subsp. enterica serovar Choleraesuis str. SC-B67 | YP_216811 | 2.4 |
| 940 | Sugar Specific PTS family, mannose-specific enzyme IIAB | *Salmonella enterica* subsp. enterica serovar | YP_216811 | 2.8 |
| 953 | Autoinducer 2 import system substrate-binding protein LsrB | *Salmonella enterica* subsp. enterica serovar Typhimurium str. LT2 | NP_462958 | 3.4 |
| 1162 | Glutamine-binding periplasmic protein precursor | *Salmonella enterica* subsp. enterica serovar Typhi str. CT18 | CAD05277 | 2.0 |
| 1220 | NAD(P)H dehydrogenase | *Salmonella enterica* subsp. enterica serovar Typhimurium str. LT2 | NP_460092 | 2.6 |
| 1261 | 50S ribosomal subunit protein L6 | *Salmonella enterica* subsp. enterica serovar Typhi str. CT18 | CAD09161 | 3.1 |
| 1277 | Superoxide dismutase | *Salmonella enterica* subsp. enterica serovar Paratyphi A str. ATCC 9150 | AAV77363 | 2.1 |
| 1656 | Carboxysome structural protein EutM | *Salmonella enterica* subsp. enterica serovar Typhimurium str. LT2 | NP_461400 | 5.6 |
| **Higher in WT vs Δ*lppAB* Δ*msbB*** | | | | |
| 349 | Probable nitrate reductase | *Salmonella enterica* subsp. enterica serovar Typhi str. CT18 | CAD07491 | 2.3 |
| 357 | Periplasmic nitrate reductase | *Salmonella enterica* subsp. enterica serovar typhimurium LT2 | NP_461202 | 6.2 |
| 1090 | ABC transporter periplasmic binding protein MlaC | *Salmonella enterica* subsp. enterica serovar Typhi str. CT18 | CAD07827 | 6.1 |
| 1276 | Superoxide dismutase, iron | *Salmonella enterica* subsp. enterica serovar Choleraesuis str. SC-B67 | YP_216437 | 2.2 |
| 1329 | Transcription anti-termination protein | *Salmonella enterica* subsp. enterica serovar Paratyphi A str. ATCC 9150 | AAV79738 | 2.1 |
| 1492 | 50S ribosomal subunit protein L6 | *Salmonella enterica* subsp. enterica serovar Typhimurium str. LT2 | NP_463255 | 2.4 |
| **Higher in WT vs Δ*lppAB* and Δ*lppAB* Δ*msbB*** | | | | |
| 567 | Serine protease | *Salmonella enterica* subsp. enterica serovar Typhimurium | CAA38420 | 3.6-3.8 |
| 1024 | Tricarboxylic transport protein | *Salmonella enterica* subsp. enterica serovar Typhimurium str. LT2 | NP_461712 | 2.2 |
| 1156 | cAMP-regulatory Protein | *Escherichia coli* 536 | YP_671327 | 3.4-8.3 |
| 1176 | Arginine-binding periplasmic protein 2 precursor | *Salmonella enterica* subsp. enterica serovar Typhi str. CT18 | CAD05326 | 2.0-2.5 |
| 1256 | Flagellar-associated GTP-binding protein | *Borreliella bavariensis* Pbi | AAU07126 | 2.5-3.0 |
| 1373 | Esterase | *Salmonella enterica* | WP_021000185 | 2.5-4.1 |
| **Higher in Δ*lppAB* Δ*msbB* vs Δ*lppAB*** | | | | |
| 165 | DNA-directed RNA polymerase beta subunit (RpoB) | *Salmonella enterica* subsp. enterica serovar Typhimurium | AIE08112 | 8.1 |
| 188 | DNA-directed RNA polymerase beta subunit | *Salmonella enterica* subsp. enterica serovar Typhi Ty2 | NP_807130 | 39.4 |
| 256 | Iron-dependent alcohol dehydrogenase | *Salmonella enterica* subsp. enterica serovar Typhimurium str. LT2 | NP_460708 | 2.8 |
| 315 | Pyruvate dehydrogenase E1 component | *Salmonella enterica* subsp. enterica serovar Typhi str. CT18 | CAD01311 | 5.8 |
| 461 | 2',3'-cyclic nucleotide 2'-phosphodiesterase | *Salmonella enterica* subsp. enterica serovar Paratyphi A str. ATCC 9150 | AAV79958 | 3.1 |
| 594 | Rho factor | *Salmonella enterica* subsp. enterica serovar Typhimurium | CAA79852 | 7.4 |
| 649 | AraC-type DNA-binding domain-containing protein | *Vibrio vulnificus* YJ016 | WP_011081127 | 2.6 |
| 713 | TolB protein precursor | *Salmonella enterica* subsp. enterica serovar Typhi str. CT18 | CAD05210 | 2.4 |
| 726 | Glycerol-3-phosphate-binding periplasmic protein | *Salmonella enterica* subsp. enterica serovar Typhi str. CT18 | CAD08072 | 2.6 |
| 773 | N-ethylmaleimide reductase | *Salmonella enterica* subsp. enterica serovar Typhimurium str. LT2 | NP_460399 | 3.1 |
| 853 | Quinone oxireductase, NADPH dependent | *Salmonella enterica* subsp. enterica serovar Choleraesuis s | WP_001541302 | 3.9 |
| 910 | Glyceraldehyde 3-phosphate dehydrogenase A | *Salmonella enterica* subsp. enterica serovar Typhi str. CT18 | CAD02064 | 2.6 |
| 929 | Sugar Specific PTS family, mannose-specific enzyme IIAB | *Salmonella enterica* subsp. enterica serovar Choleraesuis str. SC-B67 | YP_216811 | 2.3 |
| 953 | Autoinducer 2 import system substrate-binding protein LsrB | *Salmonella enterica* subsp. enterica serovar Typhimurium str. LT2 | NP_462958 | 2.1 |
| 1083 | Ubiquinone/menaquinone biosynthesis Methyltransferase UbiE | *Salmonella enterica* subsp. enterica serovar Typhi str. CT18 | CAD07922 | 2.4 |
| 1220 | NAD(P)H dehydrogenase | *Salmonella enterica* subsp. enterica serovar Typhimurium str. LT2 | NP_460092 | 2.4 |
| **Higher in Δ*lppAB* vs Δ*lppAB* Δ*msbB*** | | | | |
| 192 | Protein chain initiation factor 2 | *Salmonella enterica* subsp. enterica serovar Typhi str. CT18 | CAD07806 | 7.0 |
| 265 | Lon protease | *Salmonella enterica* subsp. enterica serovar Typhi str. CT18 | CAD08909 | 4.4 |
| ^*^ With the highest protein score from the list of the mass spectrometry report. ^#^ Based on the normalized volumes (NVs) | | | | |
